# Impact of Femoral Morphology and Stem Design on Intraoperative Periprosthetic Femoral Fractures in Cementless Total Hip Arthroplasty

**DOI:** 10.3390/jcm15134917

**Published:** 2026-06-24

**Authors:** Min-Chang Jang, Chan-Woo Park, Sang-Jin Jeong, Alhaizaey Ahmed, Youn-Soo Park, Seung-Jae Lim

**Affiliations:** 1 Department of Orthopedic Surgery, Samsung Medical Center, College of Medicine, Sungkyunkwan University, Seoul 06351, Republic of Korea; jmc2724@naver.com (M.-C.J.); existcwp@gmail.com (C.-W.P.); alhaizaey@hotmail.com (A.A.); 2 Department of Orthopedic Surgery, Myongji Hospital, College of Medicine, Hanyang University, Goyang 10475, Republic of Korea; sangjin1990@hanmail.net; 3 Department of Orthopedic Surgery, Kangbuk Samsung Hospital, College of Medicine, Sungkyunkwan University, Seoul 03181, Republic of Korea; ysp3504@skku.edu

**Keywords:** total hip arthroplasty, intraoperative periprosthetic femoral fracture, femoral morphology, Dorr classification, femoral stem design

## Abstract

**Background**: Intraoperative periprosthetic femoral fractures (IPFF) represent a concerning and often under-recognized complication in total hip arthroplasty (THA). Although several risk factors have been reported, their association with specific femoral morphology and stem geometry has not been fully addressed. This study aimed to identify the incidence and risk factors for IPFF in THA using cementless tapered stems. **Methods**: A retrospective review was conducted on 3137 primary THAs (2622 patients) performed with cementless tapered stems at a single institution between February 2011 and August 2018. Femoral morphology was classified according to the Dorr types; A (1425 hips, 45.4%), B (1542, 49.2%), and C (170, 5.4%). Femoral stems were categorized as flat, rectangular, or quadrangular tapered designs. The occurrence of IPFF was identified through surgical records and postoperative radiographs. Multivariate regression analysis was performed to identify independent risk factors for IPFF. The mean age at THA was 55 years (range, 15–96), and 52.9% of the patients were women. **Results**: The overall incidence of IPFF was 2.2% (69 hips). Non-displaced cracks in the proximal metaphysis (58 hips, 84.1%) were the most common type of fracture. The incidence of IPFF was significantly higher in Dorr type A (3.0%) and type C (4.7%) femurs compared with type B (1.2%) femurs. Multivariate regression revealed that female sex (odds ratio [OR], 1.752; *p* = 0.032) and Dorr type A (OR, 2.898; *p* < 0.001) and type C (OR, 4.530; *p* < 0.001) were significantly associated with IPFF. Additionally, the use of quadrangular tapered stems was associated with a higher risk compared with flat tapered stems (OR, 7.382; *p* < 0.001). **Conclusions**: Dorr type A and C femurs, along with female sex and the use of quadrangular tapered stems, were significant risk factors for IPFF. Our findings suggest that preoperative consideration of individual femoral morphology and careful selection of stem design are essential to mitigate the risk of IPFF in THA using cementless tapered stems.

## 1. Introduction

Total hip arthroplasty (THA) is one of the most successful surgical procedures in orthopaedics, providing reliable pain relief and functional improvement with excellent long-term survivorship [1,2,3]. With advances in implant design and surgical techniques, cementless stems have become increasingly favored in contemporary practice [4,5,6]. Recent registry-based studies have demonstrated a clear global shift toward uncemented fixation. Moldovan et al. reported that the proportion of uncemented primary hip arthroplasties increased from 23% in 2001 to over 73% in 2024, with a consistent upward trend across all age groups, including elderly patients [7]. This widespread adoption of cementless fixation has been described as the “uncemented paradox” [4], as registry data continue to demonstrate lower revision rates with cemented fixation, particularly in elderly patients [8].

Along with this trend, periprosthetic femoral fractures (PFFs) have emerged as an increasingly important cause of THA failure. The incidence of PFF after THA has nearly doubled since 2010, with an annual growth rate of 7.05% reported in a large national database study involving more than 500,000 THAs [9]. Cementless fixation has been associated with a substantially higher risk of periprosthetic femoral fracture compared with cemented fixation [8,10]. Among cementless stems, an even higher incidence of fracture has been reported with tapered wedge stems compared to fit-and-fill or cylindrical stems [11,12,13]. Tapered wedge stems achieve primary fixation through metaphyseal press-fit engagement and provide excellent initial stability and bone preservation. However, excessive hoop stress generated during femoral broaching or stem insertion may increase the risk of intraoperative fracture [14].

Intraoperative periprosthetic femoral fractures (IPFFs), which occur in approximately 1% to 2% of primary THAs and are more common with cementless fixation [15,16], may lead to fracture propagation, compromised implant stability, and an increased risk of femoral revision [17,18,19]. IPFFs are often caused by an increased hoop stress within the medullary canal during femoral preparation or stem insertion. Previous studies have identified several patient-related risk factors for IPFF, including female sex, advanced age, and poor bone quality [15,20,21]. Femoral morphology, as classified by the Dorr system, has also been recognized as an important risk factor for periprosthetic femoral fractures, particularly in Dorr type C femurs characterized by thin cortices, a widened “stovepipe” femoral canal, and poor bone quality [22]. In addition to femoral morphology, stem geometry may also influence the risk of IPFF. Even among tapered wedge stems, differences in geometric design may alter stress distribution during broaching and stem insertion [14]. Previous studies have suggested that stem geometry may influence the risk of periprosthetic femoral fracture; however, the available evidence has primarily focused on postoperative fractures, with inconsistent findings among different stem designs [12,23,24]. In contrast, the influence of stem geometry on fractures occurring during femoral preparation and stem implantation remains poorly understood. Given that both femoral morphology and stem geometry may affect stress distribution during implantation, further investigation is needed to clarify their respective roles in the development of IPFF.

Therefore, the purpose of this study was to evaluate the incidence and identify risk factors for IPFF in primary THA using cementless tapered stems. We hypothesized that both femoral morphology and stem geometry would significantly influence the risk of IPFF. Demographics, femoral morphology, and femoral stem types were compared between the groups with and without IPFF, and multivariate regression analysis was performed to identify independent risk factors for IPFF.

## 2. Materials and Methods

### 2.1. Patient Cohort

**Figure 1:**
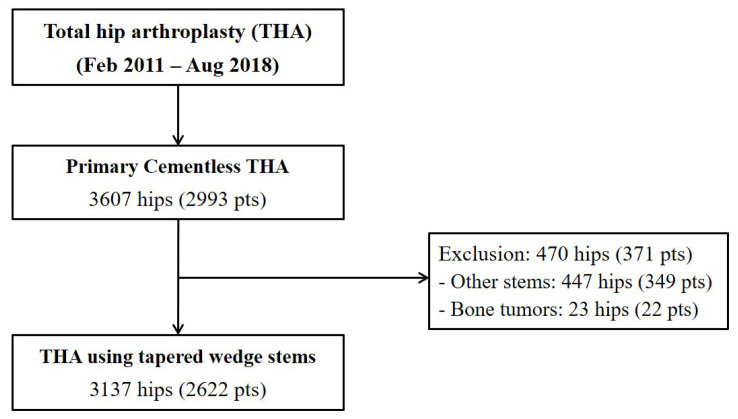
Flow diagram of the study population.

Institutional Review Board approval was obtained prior to this study. We retrospectively reviewed patients who underwent primary cementless total hip arthroplasty (THA) at a single institution between February 2011 and August 2018. A total of 3607 hips (2993 patients) were identified. The exclusion criteria were as follows: (1) revision THA; (2) use of non–tapered wedge stems (e.g., fit-and-fill, conical, cylindrical, calcar-guided short, or modular stems); and (3) bone tumors or pathologic fractures. After applying these criteria, 470 hips (371 patients) were excluded, leaving 3137 hips (2622 patients) that underwent THA using tapered wedge stems for the final analysis (Figure 1). The unit of analysis was the hip. The study cohort comprised 3137 hips from 2622 patients, including 2107 unilateral cases and 515 patients who underwent bilateral THA.

The mean age at the time of THA was 55.1 years (range, 15–96), and 47.1% of the patients were male (1476 of 3137) (Table 1). The mean body mass index (BMI) was 24.5 kg/m^2^ (range, 14.3–45.2). Indications for THA included osteonecrosis (1567 hips, 50.0%), osteoarthritis (1353 hips, 43.1%), hip fracture (155 hips, 4.9%), and inflammatory arthritis (62 hips, 2.0%). Analysis of femoral morphology according to the Dorr classification [25] showed that type B was the most common (1542 hips, 49.2%), followed by type A (1425 hips, 45.4%) and type C (170 hips, 5.4%). Rectangular tapered stems (type B1) were most frequently used (1997 hips, 63.7%), followed by flat tapered stems (type A) (810 hips, 25.8%) and quadrangular tapered stems (type B2) (330 hips, 10.5%). The distribution of stem types differed significantly according to the underlying diagnosis (χ^2^ = 163.5, df = 6, *p* < 0.001; Supplementary Table S1). Quadrangular tapered stems were used less frequently in hip fracture patients (4.5%) than in patients with osteoarthritis (8.5%) or osteonecrosis (12.9%).

### 2.2. Surgical Technique

**Figure 2:**
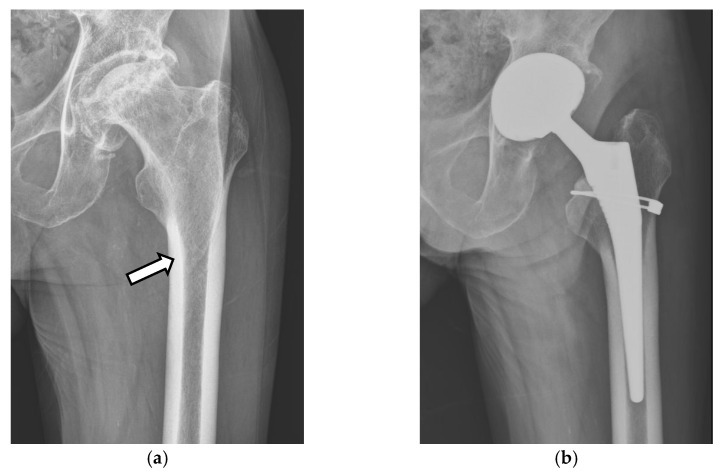
Preoperative and postoperative radiographs of a 78-year-old male with osteonecrosis of the femoral head treated with total hip arthroplasty using a type B2 quadrangular tapered stem. (**a**) Preoperative radiograph demonstrating Dorr type A femoral morphology with a narrow femoral canal and thick cortices (arrow). (**b**) Postoperative radiograph showing well-fixed stem with cerclage wiring.

All THAs were performed by three experienced arthroplasty surgeons using the modified Watson–Jones anterolateral approach. Preoperative templating was routinely performed using standard hip radiographs with magnification markers. Femoral stem selection was based on the surgeons’ preference. Femoral preparation was performed using a sequential broaching technique until appropriate insertion depth and primary stability were achieved. Intraoperative femoral fractures were managed according to the fracture pattern. Cerclage wiring was applied in all cases of IPFF (Figure 2); additional fixation methods, including cortical strut allografts or plate fixation, were employed as needed based on fracture extent and stability.

### 2.3. Implant Characteristics

A total of five different stem designs were included in this study (Table 2): Bencox II (Corentec, Cheonan, Republic of Korea), Tri-lock (DePuy Synthes, Warsaw, IN, USA), Bencox ID (Corentec, Cheonan, Republic of Korea), Taperloc Microplasty (Zimmer Biomet, Warsaw, IN, USA), and Corail (DePuy Synthes, Warsaw, IN, USA). All stems were tapered wedge designs made of a titanium alloy. According to the classification system proposed by Radaelli et al. [26], stems were categorized into three groups: type A (flat tapered stems: Tri-lock, Bencox ID, and Taperloc Microplasty), type B1 (rectangular tapered stems: Bencox II), and type B2 (quadrangular tapered stems: Corail). A collarless version of the Corail stem was used in all cases.

### 2.4. Evaluation

All clinical data were collected using an in-house electronic database. Patient demographic variables, including age, sex, BMI, and the diagnosis at the time of THA, were obtained. Femoral morphology was classified according to the Dorr classification based on preoperative anteroposterior radiographs. Classification was performed by a single experienced hip arthroplasty surgeon using the established morphological characteristics of the proximal femur, including cortical thickness and femoral canal geometry, as originally described by Dorr et al. [27]. The occurrence of IPFF was identified from operative records and intraoperative fluoroscopic or postoperative radiographic findings. IPFF were classified according to the Vancouver classification system based on fracture location, stem stability, and surrounding bone quality. Patient characteristics, femoral geometry (Dorr type), and femoral stem types were compared between patients with and without IPFF.

### 2.5. Data Analyses

Categorical variables were compared using the chi-squared test or Fisher’s exact test. Normality of continuous variables was assessed using the Shapiro–Wilk test. Continuous variables were compared using Student’s *t*-test. Multivariate logistic regression analysis was performed to identify possible risk factors for IPFF. Model calibration was assessed using the Hosmer–Lemeshow goodness-of-fit test, and model explanatory power was evaluated using the Nagelkerke R^2^ statistic. All analyses were performed using SPSS version 27 (IBM Corp., Armonk, NY, USA). Statistical significance was set at a *p* < 0.05.

## 3. Results

### 3.1. Incidence of Intraoperative Periprosthetic Femoral Fracture

The overall incidence of IPFF was 2.2% (69 of 3137 hips). Intraoperative fractures were further categorized according to the Vancouver classification (Table 3). Type A2 fractures accounted for 58 cases (84.1%), followed by type B2 fractures (5 hips, 7.2%). Type A3 and type B3 fractures were observed in three (4.3%) and two (2.9%) hips, respectively. Type B1 fracture was identified in one hip (1.4%). Subsequent postoperative PFFs occurred in three hips (4.3%) with IPFFs, including one type B1 and two type B2 IPFFs. Reoperation was required in 4 hips (5.8%), including one type B1, two type B2, and one type B3 IPFFs. Revision arthroplasty was performed in two hips (2.9%), consisting of one type B1 and one type B2.

### 3.2. Demographic Comparison Between the IPFF and Non-IPFF Groups

A significant difference was found in sex between the IPFF and non-IPFF groups (*p* = 0.039), with a lower proportion of male patients in the IPFF group (Table 4). Femoral geometry and femoral stem type showed significant differences between the groups (*p* < 0.001). The IPFF group had a higher proportion of type A and type C femurs, whereas type B femurs were more common in the non-IPFF group. In addition, type B2 stems were more frequently used in the IPFF group. There were no significant differences in age, BMI, and diagnosis at the time of THA between the 2 groups.

### 3.3. Risk Factors for IPFF

Multivariate logistic regression analysis identified several factors associated with IPFF (Table 5). Female sex was associated with an increased risk of IPFF (odds ratio [OR], 1.752; 95% confidence interval [CI], 1.051–2.921; *p* = 0.032). Femoral geometry was significantly associated with IPFF, with higher risks observed in Dorr type A (OR, 2.898; 95% CI, 1.649–5.095; *p* < 0.001) and type C femurs (OR, 4.530; 95% CI, 1.902–10.787; *p* < 0.001) compared with type B femurs. Regarding femoral stem type, type B2 quadrangular tapered stems were associated with a significantly higher risk of IPFF (OR, 7.382; 95% CI, 3.513–15.515; *p* < 0.001) compared with type A flat tapered stems. The multivariate logistic regression model demonstrated acceptable calibration according to the Hosmer–Lemeshow goodness-of-fit test (*p* = 0.642), with a Nagelkerke R^2^ value of 0.114.

## 4. Discussion

In this study, the overall incidence of IPFF was 2.2% in primary THA using cementless tapered stems. This incidence is consistent with the 2.4–3.0% range of IPFF reported in other studies of primary THA using cementless stems [15,16,28]. Although most IPFFs in this series were managed successfully with cerclage wiring, clinically significant complications still occurred in a subset of patients, with reoperation and revision arthroplasty rates of 5.8% and 2.9%, respectively. Notably, all revision cases involved Vancouver type B fractures. We identified female sex, Dorr type A and C femurs, and the use of quadrangular tapered stems as significant risk factors for IPFF.

Periprosthetic fractures are serious complications following THA, with 1-year mortality rates of 21–24% and infection rates of 5–7% reported in a United States Medicare study [29]. Moreover, the incidence of these fractures has been increasing over time; Lamb et al. [30] reported that PFF is one of the most common reasons for major reoperation following modern THA.

Although most previous studies have focused on postoperative PFF, intraoperative fractures are also clinically important, as they may lead to fracture propagation, compromised implant stability, and an increased risk of subsequent complications. Lamb et al. [21] reported that IPFF increased the risk of revision for aseptic loosening up to 7.2-fold and for subsequent postoperative PFF up to 4.3-fold compared with matched controls, with 90-day mortality increasing 1.7- to 4.0-fold. Similarly, Forlenza et al. [17] demonstrated that patients with IPFF had a 2.3-fold higher risk of all-cause revision and a 3.0-fold higher risk of aseptic loosening at 2-year follow-up.

Cementless fixation currently accounts for over 92% of primary THAs in the United States [6]. However, this widespread adoption has been accompanied by increasing concern regarding periprosthetic fractures. Cementless fixation is associated with a 2.7-fold higher risk of IPFF compared with cemented fixation [15]. The increased fracture risk is inherently related to the press-fit mechanism. Jasty et al. [31] demonstrated that even 0.5 mm of press-fit generated up to 2000 microstrain, and 1 mm of oversizing produced cortical fractures. However, this mechanism may predispose the femur to fracture particularly in cases of mismatch between femoral morphology and stem geometry. Despite these concerns, limited data have specifically evaluated the risk of intraoperative fractures according to detailed stem design and femoral morphology, which was the primary focus of the present study.

In the present study, femoral morphology was significantly associated with the occurrence of IPFF. The incidence of IPFF was higher in Dorr type A and C femurs compared with type B femurs. Dorr type A femurs, characterized by a narrow canal, may be exposed to increased hoop stress during broaching and press-fit fixation [31]. Although Dorr type A femurs generally have thicker cortices and better bone quality than Dorr type B or C femurs, their narrow metaphyseal canal may increase the risk of excessive press-fit during broaching and stem insertion. In addition, possible stem oversizing and the increased thickness associated with implant coating may further contribute to tighter implant engagement and greater cortical stress. Consequently, excessive hammering during femoral preparation or final stem implantation may increase the risk of intraoperative fracture despite the favorable bone stock. Park et al. [25] reported that Dorr type A femurs had lower stem survivorship than type B femurs, with periprosthetic fracture accounting for 67% of revision cases. Hartford et al. [32] similarly demonstrated that a smaller Dorr ratio was associated with increased odds of intraoperative fracture. On the other hand, Dorr type C femurs, with thin cortical bone and poor bone quality, are also highly vulnerable to fracture. Gromov et al. [33] reported a 5.2-fold increased risk of periprosthetic fracture in Dorr type C compared with type B femurs, and a recent meta-analysis confirmed Dorr type C as a significant risk factor with a pooled odds ratio of 4.23 [22]. Notably, while most previous studies have identified Dorr type C as the primary morphological risk factor for IPFF, the present study demonstrated that both Dorr type A and C femurs were associated with a higher incidence of IPFF. This suggests that both extremes of femoral canal morphology—a narrow canal with thick cortices and a wide canal with thin cortices—may increase the risk of intraoperative fracture through distinct biomechanical mechanisms.

Femoral stem design was another important factor influencing the risk of IPFF. In this study, quadrangular tapered stems were associated with a significantly higher risk compared with flat tapered stems. The influence of stem design on the risk of IPFF has been recognized. LeBrun et al. [24] reported that quadrangular tapered stems had the highest intraoperative fracture rate (0.92%) among four cementless stem morphologies in a cohort of 23,448 primary THAs. The geometric characteristics of quadrangular tapered stems, including angular corners and reduced conformity to the native femoral canal, may act as stress risers during implantation and thereby increase the risk of intraoperative fracture [14,34]. Quadrangular stems are less tapered in the sagittal plane with greater distal anteroposterior thickness compared to flat tapered stems. These geometric features result in tighter three-point fixation in the sagittal plane, thereby generating greater hoop stress within the medullary canal.

These findings appear to contrast with a recent meta-analysis by Borazjani et al. [23], which reported that quadrangular tapered stems were associated with a significantly lower risk of postoperative PFF (OR, 0.78; 95% CI, 0.64–0.96). This discrepancy may reflect differences in the timing and mechanism of PFFs. Intraoperative fractures are primarily related to press-fit insertion forces and local cortical stress concentration, where the angular geometry of quadrangular stems may act as a stress riser. In contrast, postoperative fractures are more influenced by implant stability and load distribution under physiological loading conditions. Once securely fixed, these same geometric features may provide enhanced rotational stability through multi-point cortical contact, thereby reducing the risk of late PFFs. These findings suggest that fracture risk may differ substantially between intraoperative and postoperative settings. Therefore, stem designs that increase local stress during implantation may not necessarily confer a higher risk of postoperative fracture and may, in some cases, provide biomechanical advantages once osseointegration has been achieved. However, differences in follow-up duration, patient populations, implant selection, and surgical techniques may also contribute to the observed discrepancies among studies.

Patient-related factors also contributed to the occurrence of IPFF. In this study, female sex was associated with an increased risk of IPFF. This finding is consistent with multiple large-scale studies. Brüggemann et al. [15] reported a 1.8-fold increased risk of IPFF in females (relative risk, 1.8; 99% CI, 1.5–2.1) in 218,423 primary THAs, and Lamb et al. [21] similarly identified female gender as a significant risk factor for IPFF in 793,823 primary THAs. Abdel et al. [16] also found that intraoperative fractures were significantly more common in females, particularly those over 65 years of age. This increased susceptibility may be related to lower bone mineral density and higher prevalence of osteoporosis in female patients [35]. Previous studies have identified osteoporosis as an important risk factor for periprosthetic femoral fractures following cementless THA [20]. Although bone mineral density was not routinely assessed in the present cohort, future studies incorporating standardized bone quality assessment may further clarify its contribution to the risk of IPFF.

These findings highlight the importance of careful preoperative planning. Assessment of femoral morphology and patient-related factors, along with appropriate selection of stem design, may help reduce the risk of IPFF. In particular, caution should be exercised when using quadrangular tapered stems in patients with Dorr type A or C femurs or in female patients.

From a clinical perspective, the present findings may help guide stem selection and surgical strategy in patients at high risk for IPFF. In patients with Dorr type A or C femoral morphology, careful femoral preparation and broaching are essential to avoid excessive hoop stress during stem insertion. Surgeons should avoid excessive press-fit fixation and consider prophylactic cerclage wiring in selected high-risk femurs. Particular caution may be warranted when using quadrangular tapered stems, which demonstrated the highest risk of IPFF in the present study. Stem selection should be individualized according to femoral morphology and bone quality. For elderly patients with poor bone stock or markedly osteoporotic femurs, alternative fixation strategies, including cemented stems, may be considered in selected cases.

Future studies should further investigate the influence of stem geometry on three-dimensional femoral torsional and bending behavior. Because stem geometry may affect load transfer and stress distribution beyond the coronal plane, advanced imaging techniques and computational biomechanical analyses may help clarify the mechanisms underlying IPFF and optimize stem selection in different femoral morphologies.

This study has several limitations. First, it was a retrospective study conducted at a single institution, which may introduce selection bias. Second, several additional radiographic parameters, including canal fill ratio, stem alignment, native femoral anteversion, stem anteversion, and version mismatch, were not analyzed and may have influenced the risk of IPFF. Third, stem selection was based on surgeon preference rather than a standardized protocol. Consequently, the distribution of stem types was not uniform and may have been influenced by patient characteristics, femoral morphology, diagnosis, and temporal changes in implant usage. Although diagnosis was included in the multivariate analysis, residual selection bias and confounding by indication cannot be completely excluded. Fourth, stem classification was based primarily on overall stem geometry. Although stems were grouped according to their geometric characteristics, implant-specific factors such as surface coating, sizing increments, broaching philosophy, and metaphyseal fit were not separately evaluated. In particular, the quadrangular tapered stem category consisted of a single implant design, making it difficult to completely distinguish the effects of stem geometry from implant-specific characteristics. Finally, occult intraoperative fractures that were not recognized clinically or radiographically may have been missed, potentially leading to underestimation of the true incidence of IPFF.

## 5. Conclusions

Dorr type A and C femurs, along with female sex and the use of quadrangular tapered stems, were significant risk factors for IPFF in cementless THA. Our findings suggest that preoperative consideration of individual femoral morphology and patient-related factors, along with careful selection of stem design, are essential to mitigate the risk of IPFF in THA using cementless tapered stems.

## Figures and Tables

**Table 1:** Baseline Characteristics.

Characteristics	*n* = 3137 (%)
Ages (years) *	55.1 (15–96)
Sex (Male)	1476 (47.1)
Body mass index (kg/m^2^) *	24.5 (14.3–45.2)
Diagnosis at primary THA	
Osteoarthritis	1353 (43.1)
Osteonecrosis	1567 (50.0)
Hip fracture	155 (4.9)
Inflammatory arthritis	62 (2.0)
Femoral geometry (Dorr type)	
Type A	1425 (45.4)
Type B	1542 (49.2)
Type C	170 (5.4)
Femoral stem type	
Type A flat tapered stems	810 (25.8)
Type B1 rectangular tapered stems	1997 (63.7)
Type B2 quadrangular tapered stems	330 (10.5)

* Values are presented as means, with ranges in parentheses. Other values are given as number of hips, with percentages in parentheses. THA, total hip arthroplasty.

**Table 2:** Details on Femoral Stems Used.

Type of Femoral Stem	*n =* 3137 (%)
Type A flat tapered stems	
Tri-lock (DePuy Synthes)	472 (15.0)
Bencox ID (Corentec)	182 (5.8)
Taperloc Microplasty (Zimmer Biomet)	156 (5.0)
Type B1 rectangular tapered stems	
Bencox II (Corentec)	1997 (63.7)
Type B2 quadrangular tapered stems	
Corail (DePuy Synthes)	330 (10.5)

Values are given as the number of hips, with percentages in parentheses.

**Table 3:** Classification of Intraoperative Periprosthetic Femoral Fractures.

Characteristic	Number of IPFF (*n* = 69)	Postop PFF (*n* = 69)	Any Reoperation (*n* = 69)	Revision (*n* = 69)
Vancouver type				
A1	0 (0%)			
A2	58 (84.1%)			
A3	3 (4.3%)			
B1	1 (1.4%)	1 (1.4%)	1 (1.4%)	1 (1.4%)
B2	5 (7.2%)	2 (2.9%)	2 (2.9%)	1 (1.4%)
B3	2 (2.9%)		1 (1.4%)	
C	0 (0%)			

Values are given as the number of hips, with percentages in parentheses. IPFF, intraoperative periprosthetic femoral fracture; PFF, periprosthetic femoral fracture.

**Table 4:** Comparison between IPFF and Non-IPFF Groups.

Characteristic	IPFF (*n* = 69)	Non-IPFF (*n* = 3068)	*p*-Value
Ages (years) *	56.0 (19–90)	55.1 (15–96)	0.597
Sex (male)	24 (34.8)	1452 (47.3)	0.039
Body mass index (kg/m^2^) *	24.2 (16.7–32.8)	24.5 (14.3–45.2)	0.571
Diagnosis at primary THA			0.425
Osteoarthritis	31 (44.9)	1322 (43.1)	
Osteonecrosis	31 (44.9)	1536 (50.1)	
Hip fracture	6 (8.7)	149 (4.9)	
Inflammatory arthritis	1 (1.4)	61 (2.0)	
Femoral geometry (Dorr type)			<0.001
Type A	43 (62.3)	1382 (45.0)	
Type B	18 (26.1)	1524 (49.7)	
Type C	8 (11.6)	162 (5.3)	
Femoral stem type			<0.001
Type A flat tapered stems	10 (14.5)	800 (26.1)	
Type B1 rectangular tapered stems	32 (46.4)	1965 (64.0)	
Type B2 quadrangular tapered stems	27 (39.1)	303 (9.9)	

* Values are presented as means, with ranges in parentheses. Other values are given as number of hips, with percentages in parentheses. IPFF, intraoperative periprosthetic femoral fracture; THA, total hip arthroplasty.

**Table 5:** Multivariate Regression Analysis for IPFF.

Characteristic	Adjusted OR	95% CI	*p*-Value
Ages (years)	0.997	0.979–1.015	0.725
Sex (female)	1.752	1.051–2.921	0.032
Body mass index (kg/m^2^)	0.973	0.907–1.043	0.434
Diagnosis at primary THA			
Osteoarthritis	1.00		
Osteonecrosis	0.961	0.547–1.686	0.889
Hip fracture	2.430	0.907–6.508	0.077
Inflammatory arthritis	0.579	0.074–4.506	0.601
Femoral geometry (Dorr type)			
Type A	2.898	1.649–5.095	<0.001
Type B	1.00		
Type C	4.530	1.902–10.787	<0.001
Femoral stem type			
Type A flat tapered stems	1.00		
Type B1 rectangular tapered stems	1.137	0.554–2.335	0.726
Type B2 quadrangular tapered stems	7.382	3.513–15.515	<0.001

IPFF, Intraoperative periprosthetic femoral fracture; OR, odds ratio; CI, confidence interval; THA, total hip arthroplasty.

## Data Availability

The datasets used and analyses are available from the corresponding authors upon reasonable request.

## Supplementary Materials

The following supporting information can be downloaded at https://www.mdpi.com/article/10.3390/jcm15134917/s1, Table S1. Distribution of Femoral Stem Types According to Diagnosis at Primary Total Hip Arthroplasty (*n* = 3137).

## Abbreviations

The following abbreviations are used in this manuscript:

THA	Total hip arthroplasty
PFF	Periprosthetic femoral fracture
IPFF	Intraoperative periprosthetic femoral fracture
BMI	Body mass index
OR	Odds ratio
CI	Confidence interval
